# “Closer‐to‐home” strategy benefits juvenile survival in a long‐distance migratory bird

**DOI:** 10.1002/ece3.5395

**Published:** 2019-07-23

**Authors:** Yachang Cheng, Wolfgang Fiedler, Martin Wikelski, Andrea Flack

**Affiliations:** ^1^ Department of Migration Max Planck Institute of Animal Behavior Radolfzell Germany; ^2^ Department of Biology University of Konstanz Konstanz Germany; ^3^ Centre for the Advanced Study of Collective Behaviour University of Konstanz Konstanz Germany

**Keywords:** bio‐logging, long‐distant migration, migration strategy, ODBA, survival

## Abstract

Human‐induced changes in the climate and environment that occur at an unprecedented speed are challenging the existence of migratory species. Faced with these new challenges, species with diverse and flexible migratory behaviors may suffer less from population decline, as they may be better at responding to these changes by altering their migratory behavior. At the individual level, variations in migratory behavior may lead to differences in fitness and subsequently influence the population's demographic dynamics. Using lifetime GPS bio‐logging data from 169 white storks (*Ciconia ciconia*), we explore whether the recently shortened migration distance of storks affects their survival during different stages of their juvenile life. We also explore how other variations in migratory decisions (i.e., time, destination), movement activity (measured using overall body dynamic acceleration), and early life conditions influence juvenile survival. We observed that their first autumn migration was the riskiest period for juvenile white storks. Individuals that migrated shorter distances and fledged earlier experienced lower mortality risks. In addition, higher movement activity and overwintering “closer‐to‐home” (with 84.21% of the tracked individuals stayed Europe or North Africa) were associated with higher survival. Our study shows how avian migrants can change life history decisions over only a few decades, and thus it helps us to understand and predict how migrants respond to the rapidly changing world.

## 1 INTRODUCTION

Migration is a ubiquitous phenomenon that has evolved as an adaptation to seasonally changing environments (Newton, 2008). But within the same species, or even population, we often observe tremendous variation in specific migration features (Chapman, Brönmark, Nilsson, & Hansson, 2011; Flack et al., 2016). This may be the outcome of trade‐offs between the costs and benefits of different migration strategies, leading to differences in individual fitness, and subsequently influencing demographic dynamics (Lok, Overdijk, & Piersma, 2015; Palacín, Alonso, Martín, & Alonso, 2017). Life in the Anthropocene is challenging many ancient evolutionary adaptations including migration. Selection pressures change quickly, favoring individuals that are better adapted to human‐modified landscapes (Otto, 2018). These human‐shaped selection pressures are caused by hunting, poisoning, electrocution, food subsidies or global habitat and climate change, and many more (Oro, Genovart, Tavecchia, Fowler, & Martínez‐Abraín, 2013; Otto, 2018), and may indeed force migrants to respond quickly to survive. In recent years, numerous studies have shown that birds alter their migratory behavior by changing their routes and/or timing, dynamically altering their wintering strategies, or even becoming residents (Fiedler, 2003; Palacín et al., 2017; Visser, Perdeck, Balen, & Both, 2009). Species with more diverse migratory behavior or higher overall flexibility seem to suffer less from population decline as they are better at adjusting to these changes (Gilroy, Gill, Butchart, Jones, & Franco, 2016; Saino et al., 2011), and human‐altered selection forces might favor individuals with certain behavioral or conditional traits (Otto, 2018). Thus, if an individual's behavioral traits or its decisions during ontogeny allow us to explain patterns of longevity, we will gain insights into basic life history evolution and understand how migrants respond to the changing world. It will also allow us to develop a toolset for conservation managers to anticipate changes in populations of wild birds.

One frequently studied migrant is the white stork *Ciconia ciconia*. Many studies provide detailed knowledge on this widely distributed, opportunistic species: European white storks were exclusively migratory, but in the last three decades, it has been reported that the subpopulation that migrates on the western flyway between Central Europe, Iberia, and western Africa is shortening its migratory distances. New wintering and resident behavior emerged on the Iberian Peninsula (Tortosa, Manez, & Barcell, 1995), resulting in larger numbers of wintering storks in Europe (Arizaga et al., 2018). New and stable food sources like open landfills or the invasive red swamp crayfish *Procambarus clarkii* may be contributing to the resident behavior of storks (Gilbert et al., 2016; Tortosa, Caballero, & Reyes‐López, 2002). In addition, wintering in Europe may require less energy in terms of movement costs and foraging efforts (Flack et al., 2016; Rotics et al., 2017). Thus, the question emerges: is long‐distance migration in white storks decreasing or even disappearing? Current studies which investigated the relationship between changes in migration strategy and breeding performance were inconsistent (Gilbert et al., 2016; Massemin‐Challet et al., 2006). Here we hypothesize that these current changes in the white stork's migratory behavior will be reflected in their overall fitness (i.e., individuals with shorter migratory distance have higher survival), and thereby influence the entire population's demography.

Like in many long‐lived birds, the first year of life is the most challenging period for a stork (Rotics et al., 2016; Sergio et al., 2011). Thus, due to this high mortality, we expect a strong selective pressure on white stork juveniles with differing traits. Thanks to technical and methodological improvements in bio‐logging, we now have the opportunity to reveal the relationship between migratory decisions and fitness consequences in the context of global changes (Kays, Crofoot, Jetz, & Wikelski, 2015; Wilmers et al., 2015). And because bio‐logging devices also have integrated sensors that monitor animal movement activity precisely, we may also gain insights into the birds' internal states and processes (Wilson et al., 2014). Here, we used the lifetime tracks of 169 white stork juveniles to link migratory decisions (i.e., time, distance, and destination), movement activity, and individual traits to their subsequent survival probabilities.

## 2 METHODS

### 2.1 Dataset

From 2013 to 2017, we equipped a total of 193 juvenile white storks from five different regions in Germany and Austria with solar GPS‐ACC loggers (e‐obs GmbH). The total weight of transmitter and harness was 66 g, corresponding to approximately 2% of the mean body mass of white storks (for details see Flack et al., 2016). All tags recorded GPS locations and three‐dimensional body acceleration for 18 hr a day (between 2:00 and 20:00 UTC). GPS positions were recorded at intervals ranging from 1 s to 20 min; three‐axial body acceleration data (hereafter ACC) were collected in short bursts (lengths ranging from 1.2 to 4.1 s) every 0.5–10 min at 10.54–33.33 Hz. Data were stored on the device until downloaded via an ultra‐high‐frequency radio link or sent via the mobile phone network. We excluded (a) 13 of the tagged chicks that did not fledge; (b) five individuals from Bavaria which migrated along the eastern migration route (i.e., through Eastern Europe and Israel to East Africa); (c) five individuals with broken tags, or a too tight harness (which we identified after removing the harness of the captured bird); and (d) one bird that departed unusually late (departed at day of the year 277, population mean ± *SD*: 228.824 ± 11.120). Overall, 169 fledglings were included in this study, see details of tagging year and location in Appendix S1: Table S1. Permits for tagging and tracking were issued by the authorities of the corresponding Federal States of Germany.

### 2.2 Hazard factors influencing survival

We examined the effect of the following migration decisions on survival: departure date, movement distances, wintering region, and overall movement activity. Migration departure date was defined as the first day with a latitudinal difference of −0.38°, representing a southward movement of approximately 50 km. We calculated the daily displacement distances (hereafter daily distance) as the distance between the first GPS locations of two consecutive days. Daily distances were log‐transformed. Wintering region was defined as the most southern point of the birds' first winter and grouped in three categories, that is, Europe (north of the Strait of Gibraltar), North Africa (between Strait of Gibraltar and 20°N), and Sub‐Saharan Africa (between 12 and 20°N). Additionally, we examined how different individual traits in early life influence juvenile survival. These included hatching rank, number of siblings, sex, and fledging date (defined as the first of two consecutive days during which the distance to the nest exceeded 500 m). Fledging and departure dates were converted to day of the year (DOY, i.e., 1–366).

To delineate physical movement characteristics, we estimated movement activity levels using overall dynamic body acceleration (hereafter ODBA), often used as a proxy for energy expenditure and a potential indicator of an animal's internal state (Wilson et al., 2006). To calculate ODBA, we subtracted the daily mean of each acceleration axis from the corresponding raw values to remove the static component of ACC (Wilson et al., 2006). The sum of all three axes provided a series of ODBA values for each burst. Then we standardized the ODBA value of each burst by dividing it with the product of ACC sampling frequency and burst duration, and calculated the median ODBA per day as daily ODBA. The ODBA calculation was performed using the “moveACC” package in R v. 3.3.3 (Scharf, 2018).

### 2.3 Survival analysis

We defined three life phases to examine which factors impacted survival during these specific life history stages: postfledging period—after fledging and before migration onset; migration period—between departure date and September 30; and wintering period—between October 1 and February 28 of the following year (Rotics et al., 2017; Sanz‐Aguilar, Jovani, Melián, Pradel, & Tella, 2015). Individuals that vanished or were alive by the end of the specific time periods were censored (Therneau & Grambsch, 2013).

After visualizing GPS and ACC data, we identified an individual as dead when one of the following criteria was met: (a) accelerometer data showed a flat line for more than 24 hr; (b) GPS positions were located inside a radius of 100 m for more than three consecutive days; or (c) we observed GPS locations along, near, or inside anthropogenic structures before the animal disappeared (e.g., houses, yards, roads identified using Google Earth). We confirmed or detected mortality by localizing the carcass in the field whenever possible. For each mortality event, we defined the death day as the first stationary or motionless day. Individuals that disappeared without any death criteria being met were marked as vanished. In total, 64 individuals survived until the end of their first winter; we identified 58 deaths in the field, 21 deaths using GPS and ACC data, six instances by locating the stork near to human structures (death event criteria iii), and 20 storks vanished due to unknown reasons. We had no incidents of birds losing their loggers. Also, we considered different tagging locations as a metapopulation and not differentiated them.

We used the Kaplan–Meier estimator (Collett, 2015) to illustrate the overall survival pattern, estimate median survival day and survival rates (since fledging) for each of the specific life history stages. To examine the relationship between survival and the above‐mentioned covariates, we used a Cox proportional hazards regression model (hereafter Cox model), as it is the most widely used multivariate survival analysis for continuous time‐to‐event data (Therneau & Grambsch, 2013). Because the covariates might influence survival differently during different life history stages, we built separate Cox models for each of the three stages. Daily ODBA and daily distances were summarized for each stage by calculating medians. We used restricted cubic splines (three degrees of freedom) to specify fledging and departure date (Harrell, 2015). We stratified tagging year to control for potential interannual variation. Because conditions in one season may influence subsequent seasons through carry‐over effects (Klaassen et al., 2014), we included stage‐specific covariates into the models of subsequent life stages. The three initial full models are described in detail in Appendix S1: Table S2.

Regularization and variable selection using Elastic Net were performed using the “glmnet” package in R v.3.3.3 (Friedman, Hastie, & Tibshirani, 2009). If any nonlinear effects were selected, we included all knots of the restricted cubic spline term. Covariates with nonzero coefficients were refitted to the model with the “rms” package, and redundant predictors were dropped (Harrell, 2015). We used likelihood ratio tests to determine the models' overall statistical significance and Wald tests to determine *p*‐values of the individual covariates. All diagnostics were performed and plotted using the “survival” and “rms” packages in R v.3.3.3 (Harrell, 2018; Therneau, 2015). Visualizations of log‐relative hazard for each covariate were adjusted by the other covariates of the model, and we used data between the 0.05 and 0.95 quantiles. Detailed information on model diagnostics can be found in Appendix S2. We found the proportional hazard assumption did not hold true for one covariate (Appendix S2: Table S2). We therefore tested the potential causes of the nonproportional hazard and added the interaction term in the final migration stage Cox model (Keele, 2010).

Finally, we determined how sensitive the models are to any potential bias caused by nonrandom censoring (Collett, 2015). Although we found that vanished birds were not randomly distributed, that is, vanished birds had a higher migration distance compared to alive birds (*t* = −3.355, *df* = 16.107, *p* = 0.004), and similar distances as dead individuals (*t* = −0.066, *df* = 21.472, *p* = 0.948), a sensitivity analysis suggested that our findings are robust to dependent censoring (see detailed discussion in Appendix S2).

## 3 RESULTS

### 3.1 Overall migration and survival pattern

**Figure 1:**
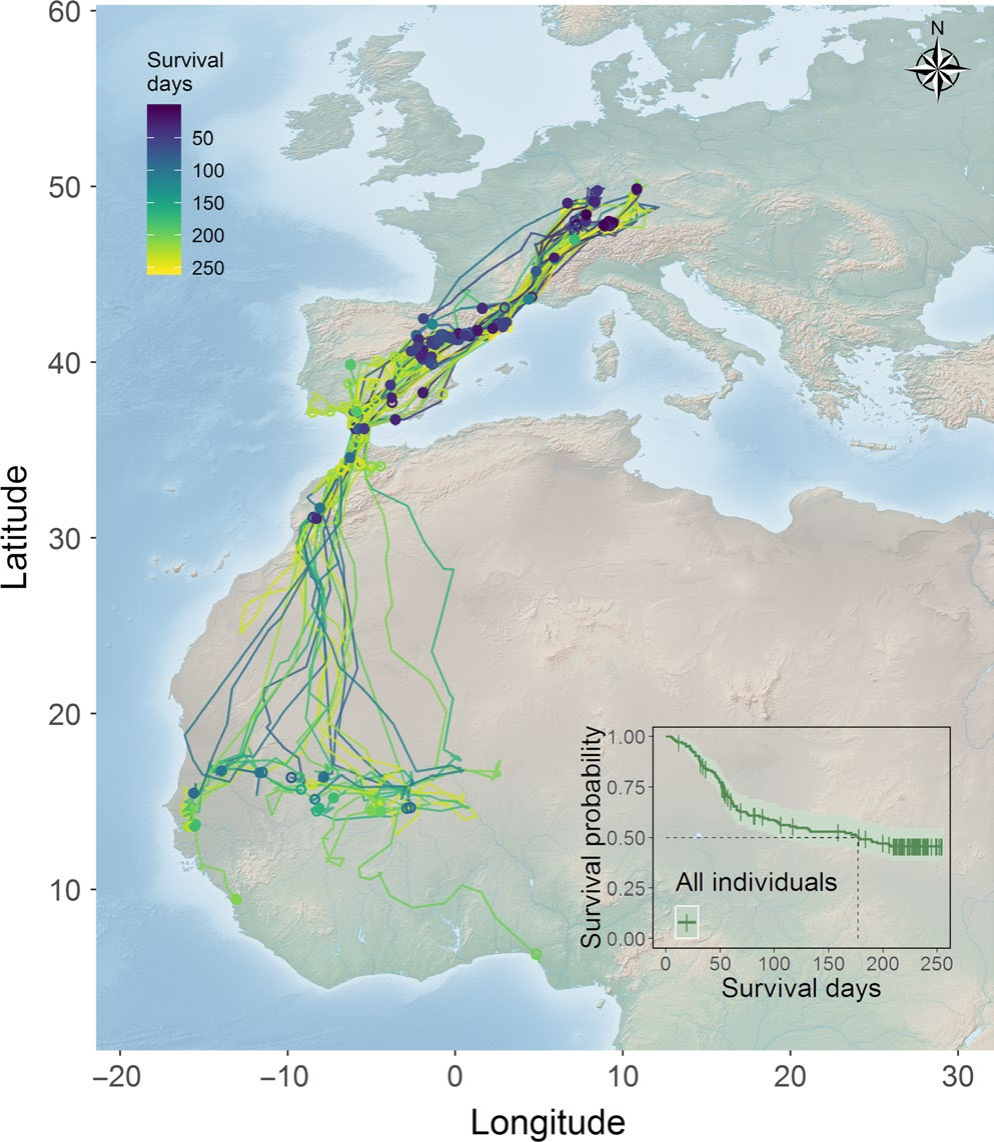
Movement trajectories of storks during the study period (i.e., postfledging time, first autumn migration, and wintering period). Filled circles indicate the death locations. Trajectories ending with open circles indicate censored individuals (alive or vanished). Trajectories are color‐coded based on the total number of survival days. The bottom right insert shows the survival curve with 95% confidence interval (shaded area). Short vertical lines indicate censored events. Dashed lines correspond to median survival time. (Background map from Natural Earth)

All juvenile white storks migrated along the well‐described western route, through the western European continent, and many crossed the Gibraltar Strait to reach finally the Sahel region or the coast of West Africa. The majority of deaths (84.88%) occurred on the European continent (Figure 1). Migration was the riskiest period for juvenile storks as it exhibited the steepest decrease in the survival probability curve (insert Figure 1, day 31–79). This sharp decrease of survival probability slowed down during winter. Cumulated survival rates since fledging for the postfledging period, migration, and winter were 0.875 (95% CI = 0.827–0.927), 0.609 (95% CI = 0.538–0.689), and 0.457 (95% CI = 0.384–0.543), respectively. Median survival time, that is, time point by which half of the storks were expected to survive, was 177 days (corresponding to early January).

### 3.2 Hazard factors

During the postfledging period, 18 of 169 storks died. However, none of the covariates influenced survival during that period significantly. Thus, we mainly focus on migration and the wintering period.

**Figure 2:**
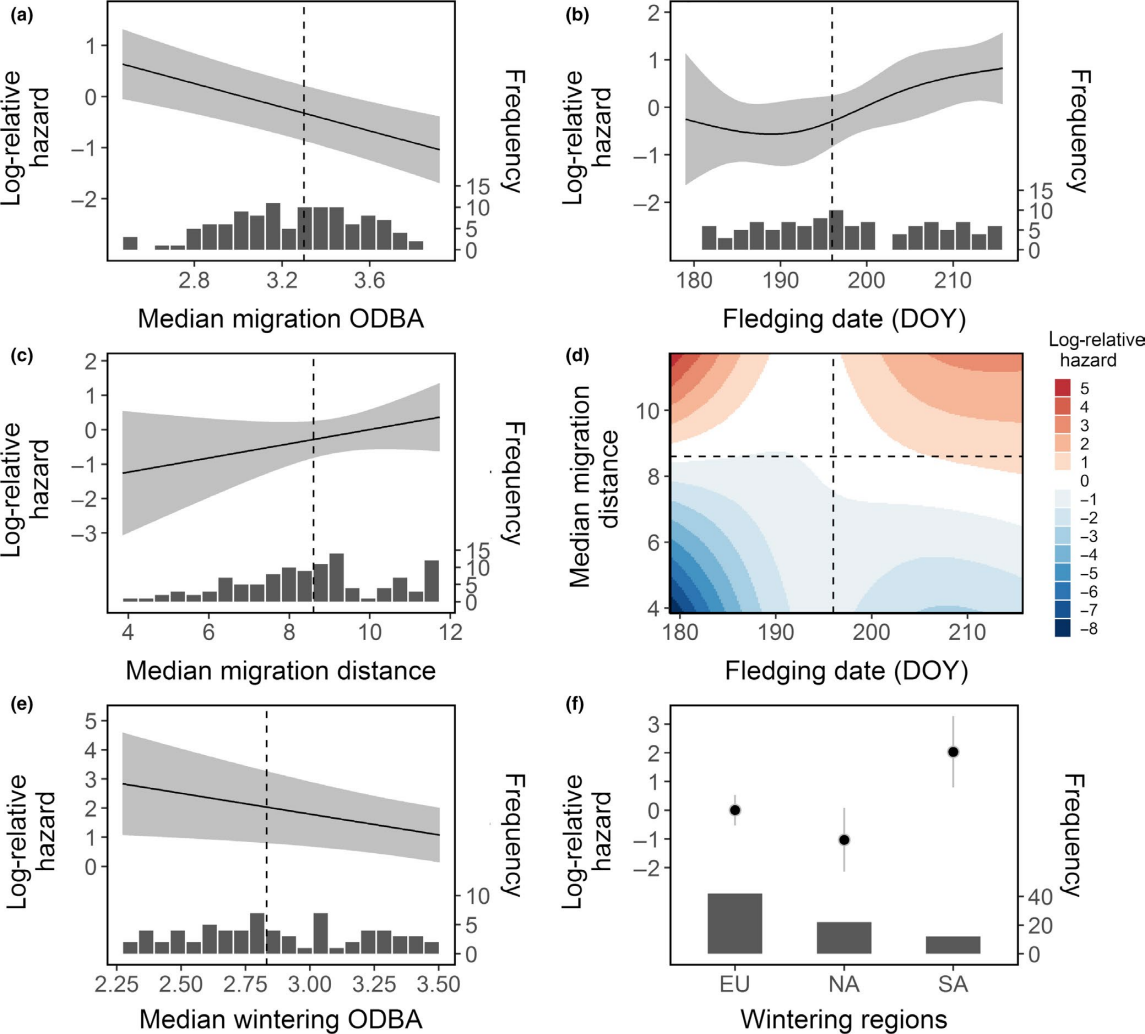
Estimated log‐relative hazard of each covariate of the Cox model for the first autumn migration (a–d) and first winter (e, f), with histogram of data distribution at the bottom: (a) median daily migration overall dynamic body acceleration (ODBA), (b) fledging date, (c) median daily migration distance, (d) interaction between fledging date and migration distance, (e) median daily wintering ODBA, and (f) wintering region, that is, Europe (EU), North Africa (NA), and Sub‐Saharan Africa (SA). Black line or points represent the predicted values adjusted by the mean or the reference level of other model covariates. Gray bands or bars correspond to the 95% confidence limit. Dashed vertical or horizontal lines show the median value of covariates. DOY‐day of the year

We found that survival during migration was influenced by daily movement activities (ODBA), daily migration distance, fledging date (as nonlinear effect), and the interaction between daily distance and fledging date (Appendix S2: Table S3, *df* = 8, *R* ^2^ = 0.453, log‐ratio test *p* = 0.000). Larger daily ODBA, shorter daily migration distance, and early fledging dates were linked to reduced mortality risks during migration (Figure 2a–c). In addition, the relationship between migration distance and mortality was influenced by fledging date (Figure 2d), showing that survival was relatively independent of migration distance around the median fledging date July 15th. Storks that fledged earlier and migrated less than the median of the population had the lowest mortality risk.

We found that during winter, median ODBA and wintering region affected juvenile survival (Appendix S2: Table S5, *df* = 3, *R* ^2^ = 0.202, log‐ratio test *p* = 0.007). Similar to migration, larger median daily ODBA reduced the mortality risk, after controlling for wintering regions (Figure 2e). Individuals overwintering in Europe and North Africa had lower mortality hazards compared to those staying in the traditional wintering grounds of Sub‐Saharan Africa (Figure 2f).

## 4 DISCUSSION

By exploring the lifetime tracks of juvenile white storks, we obtained a robust estimation of true survival rates and identified hazard factors in the early life stages of this long‐lived migrant. Our results showed that the first southward migration is the riskiest period for juvenile white storks. In agreement with our hypothesis, we found that mortality risk increases with migration distance, a finding that is in accordance with other long‐distance migrants (Klaassen et al., 2014; Lok et al., 2015). This relationship was also reflected in the effect of the chosen wintering region. Low survival probabilities of storks overwintering in Sub‐Saharan Africa may drive the overall change in migration strategy that has been observed in the last three decades. It may also explain why only a small number of our study individuals (15.79%) moved toward Sub‐Saharan Africa, whereas the majority of juveniles adopted the “closer‐to‐home” strategy, that is, terminated their migratory journey on the Iberian Peninsula (55.26%) or in North Africa (28.95%), where they experienced lower mortality rate during winter.

Even though successful storks migrate shorter distances and benefitting from sufficient food and warmer temperatures in these southerly European regions (Martín, Onrubia, & Ferrer, 2016), we observed that higher movement activities (ODBA) during migration and wintering lowered their mortality risks. Because the flight costs for soaring migrants are relatively low (Duriez et al., 2014), we suggest that high values of daily ODBA point toward more intensive foraging behavior during both the migration and wintering period. Previous studies found that storks with lower flight ODBA survive better (Rotics et al., 2016), or travel further (Flack, Nagy, Fiedler, Couzin, & Wikelski, 2018), but our findings cannot be directly linked to individual flight performance, as our metric for median daily ODBA did not only include flight but the birds' overall activities.

Like in other species (Menu, Gauthier, & Reed, 2005; Naef‐Daenzer, Widmer, & Nuber, 2001), later fledging juveniles experienced higher mortality risks. This relationship was influenced by migration distance: if individuals fledged before or after the median fledging date, their survival was influenced more strongly by migratory distance, indicating that their optimal migratory strategy was state‐dependent (Figure 2d). None of the other examined individual traits, that is, sex, hatch rank, numbers of siblings, or departure date influenced short‐term survival. This may be because storks can potentially compensate for differences in nestling body conditions later on in life (Aguirre & Vergara, 2007), although we did not observe a relationship between hatching rank and migratory distance, as suggested in that study.

As an opportunistic species, white storks exhibit high levels of behavioral flexibility and can potentially adapt relatively fast to changing environments (Cuadrado, Sánchez, Barcell, & Armario, 2016; Martín et al., 2016). In the mid‐twentieth century, droughts in the Sahel region had a strong negative effect on the western subpopulation of white stork. Although precipitation conditions gradually improved (Nevoux, Barbraud, & Barbraud, 2008), hunting pressure has increased drastically in this region (Zwarts, Bijlsma, Kamp, & Wymenga, 2012), representing a new significant threat to migrating birds. Our data show that 41.67% of the storks that overwintered in Sub‐Saharan Africa died due to hunting (five out of 12). Thus, given the effect of wintering region on survival, we expect that the number of juveniles overwintering in Europe keeps increasing. It has also been suggested that adults which overwinter closer to home or even stay completely at their breeding grounds might experience benefits during the upcoming breeding season (Arizaga et al., 2018; Rotics et al., 2018) and that migratory distance of white storks decreases with age (Fiedler, 2001). We therefore suggest that both the potential breeding benefits for adults and the higher juvenile survival contribute to the increasing number of wintering white storks in Europe and that a shortening of migratory distance could be the optimal migration strategy for the current western European white stork population.

In the past few decades, we have witnessed that white storks change their behavior as a response to human‐caused alterations in their environmental surroundings, yet the future development of these behavioral changes is unclear. The number of white storks overwintering in Portugal has already increased tenfold in the last two decades (Catry et al., 2017). It has also been documented that an increasing number of individuals of the Spanish subpopulation use their nests all year round (Gilbert et al., 2016), suggesting an intensified competition among breeders. Density‐dependent intraspecific competitions and the foreseeable closure of landfill locations (Gilbert et al., 2016; Sanz‐Aguilar et al., 2015) might cause further alterations of white stork migration and survival patterns. Although for Egypt vultures *Neophron percnopterus* no negative short‐term effects of the landfill closures have been documented (Katzenberger et al., 2017), it is hard to predict the future consequences for white storks. Their highly flexible behavior might enable them to regain a long‐distance migratory strategy once selection pressures change again. Thus, further studies are essential to investigate how environmental or social conditions influence the life history strategies of white storks from different populations (Chapman et al., 2011; Gillis, Green, Middleton, & Morrissey, 2008).

## CONFLICT OF INTEREST

None declared.

## AUTHORS' CONTRIBUTION

All authors contributed to study design, conception and gave approval for publication. W.F. and A.F collected data. Y.C and A.F conducted the analysis and wrote the manuscript in collaboration with all.

## Data Availability

The data used for this study are available through the Movebank Data Repository (https://www.movebank.org): with https://doi.org/10.5441/001/1.v1cs4nn0, https://doi.org/10.5441/001/1.c42j3js7, https://doi.org/10.5441/001/1.4192t2j4, https://doi.org/10.5441/001/1.ck04mn78, https://doi.org/10.5441/001/1.71r7pp6q (Fiedler, Flack, Schäfle, et al., 2019; Fiedler, Flack, Schmid, Reinhard, & Wikelski, 2019; Fiedler, Hilsendegen, et al., 2019; Fiedler, Leppelsack, et al., 2019; Fiedler, Niederer, Schönenberger, Flack, & Wikelski, 2019).
