# Supplementary material for: “Closer‐to‐home” strategy benefits juvenile survival in a long‐distance migratory bird
Source: Ecol Evol. 2019 Jul 23;9(16):8945–52. doi: 10.1002/ece3.5395 (PMC6706183; doi:10.1002/ece3.5395)
Supplement: Supplementary file 1 [file ECE3-9-8945-s001.pdf]

Appendix S1 Tagging details and initial full models

“Closer-to-home” strategy benefits juvenile survival in a long-distance migratory bird

Yachang Cheng, Wolfgang Fiedler, Martin Wikelski, Andrea Flack

Table S1 Sample sizes of the different stork tagging location

Region (reference location)	2013	2014	2015	2016	2017
Bavaria (49.84° N, 10.82° E), Germany	0	6	10	2	0
Oberschwaben (48.01° N, 9.02° E), Germany	0	9	7	0	0
Rheinland-Pfalz (49.22° N, 8.17° E), Germany	0	0	21	23	12
Southwest Germany (47.75° N, 8.93° E)	12	57	2	2	3
Vorarlberg (47.39° N, 9.70° E), Austria	0	0	0	3	0
Total	12	72	40	30	15

Table S2 Initial full model of Cox proportion hazard for post-fledging, migration and wintering stage.

<table>
<tr><th>Stage</th><th colspan="2">Covariates</th><th>Total df.</th></tr>
<tr><td rowspan="2">Post-fledging</td><td>Individual condition</td><td>sex, hatch rank, sibling number, fledging date</td><td rowspan="2">10</td></tr>
<tr><td>Movement</td><td>log median daily post-fledging distance, median daily post-fledging ODBA*</td></tr>
<tr><td>Migration</td><td>Individual condition</td><td>sex, hatch rank, sibling number, fledging date</td><td>15</td></tr>
</table>

	Movement	departure date, log median daily migration distances, median daily migration ODBA	
	Carry over	log median daily post-fledging distances, median daily post-fledging ODBA	
Wintering	Individual condition	sex, hatch rank, sibling number, fledging date	19
	Movement	departure date, log median wintering distances, median daily wintering ODBA, wintering regions	
	Carry over	log median daily post-fledging distances, log median daily migration distances, median daily post-fledging ODBA, median daily migration ODBA	

* ODBA: Overall Dynamic Body Acceleration
