# Supplementary material for: “Closer‐to‐home” strategy benefits juvenile survival in a long‐distance migratory bird
Source: Ecol Evol. 2019 Jul 23;9(16):8945–52. doi: 10.1002/ece3.5395 (PMC6706183; doi:10.1002/ece3.5395)
Supplement: Supplementary file 2 [file ECE3-9-8945-s002.pdf]

Appendix S2 Model matrixes, diagnoses, and sensitivity analysis

"Closer-to-home" strategy benefits juvenile survival in a long-distance migratory bird

Yachang Cheng, Wolfgang Fiedler, Martin Wikelski, Andrea Flack

To diagnose the cox proportional hazards models, we examined three aspects of their residuals (Klein and Mosechberger 2012, Xue and Schifano 2017). Firstly, for each covariate, we assessed the functional form of the covariates using martingale residuals. Except for fledging and departure dates, we assumed that the continuous covariates had a linear functional form. Secondly, we checked the proportional hazard assumption using Schoenfeld residuals with rank-transformed time (Park and Hendry 2015). Thirdly, we checked the potential outliers using deviance residual, i.e. birds that died too early or too late compared to model predictions. All diagnostics were performed and plotted using the "survival" and "rms" packages for R (Therneau 2015, Harrell 2018).

S2.1 Migration period

Fledging date (restricted cubic splines, 3 degrees of freedom), migration distance and migration ODBA (overall dynamic body acceleration) were selected for the cox model of the migration period (Table S1). We omitted the results of the graphical test of the functional form (martingale residuals) as all functional forms were correct. Table S1 and Table S2 show the model matrix and results of assessing the proportion hazard assumption. However, we found that the proportion hazard assumption did not hold for migration distance. We tested the potential cause of this non-proportion hazard and added an interaction term in the final migration period cox model (Keele 2010). The adjusted migration model matrix (Table S3) did not violate the proportion hazard assumption (Table S4).

Table S1 Migration stage cox model matrix without the interaction term.

Matrix	coef	S.E.coef	Wald Z	p
Fledging date	-0.162	0.069	-2.36	0.018*
Fledging date’	0.435	0.200	2.17	0.030*
Fledging date’’	-1.081	0.611	-1.77	0.077
Log median migration distance	0.607	0.110	5.54	0.000*
Median migration ODBA	-0.929	0.238	-3.91	0.000*
Model overall summary	N=126, number of events=39, df=5<br>R^2=0.391, log ratio test p=0.000*			

Table S2 Proportion hazard assumption test for migration stage model without interaction.

Log median migration distance violated the assumption.

	rho	chisq	p
Fledging date	0.185	1.026	0.311
Fledging date’	-0.124	0.477	0.490
Fledging date’’	0.097	0.286	0.593
Log median migration distance	-0.433	9.319	0.002*
Median migration ODBA	0.049	0.065	0.758
Global	-	10.528	0.062

Table S3 Cox survival migration stage cox model matrix with an interaction term.

Matrix	coef	S.E.coef	Wald Z	p
Fledging date	1.359	0.558	2.44	0.015*
Fledging date’	-2.821	1.329	-2.12	0.034*

Fledging date’’	7.174	3.796	1.89	0.059
Log median migration distance	31.171	10.863	2.87	0.004*
Median migration ODBA	-1.161	0.279	-4.17	0.000*
Fledging date: Log median migration distance	-0.164	0.058	-2.82	0.005*
Fledging date’: Log median migration distance	0.355	0.142	2.50	0.013*
Fledging date’’: Log median migration distance	-0.907	0.411	-2.21	0.027*
Model overall summary	N=126, number of events=39, df=8<br>R^2=0.453, log ratio test p=0.000*			

Table S4 Proportion hazard assumption test for migration stage model with interaction. No covariate violated the assumption.

	rho	chisq	p
Fledging date	-0.107	0.658	0.417
Fledging date’	-0.031	0.066	0.797
Fledging date’’	0.005	0.002	0.962
Log median migration distance	-0.106	0.627	0.428
Median migration ODBA	0.131	0.814	0.367
Fledging date: Log median migration distance	0.099	0.558	0.455
Fledging date’: Log median migration distance	-0.020	0.029	0.865
Fledging date’’: Log median migration distance	-0.015	0.019	0.892
Global	-	11.614	0.169

Deviance residuals of the migration period cox model with the interactive term (Figure S1) suggest five potential outliers which died too earlier, and one that died too late, compared to the model prediction using (-2,2) as the threshold (Klein and Mosechberger 2012). Three of

these storks died from electrocution by power lines, and two were predated. The bird that died to late in Mali had an unknown death reason. Due to the small sample sizes, we did not exclude these individuals from our model.

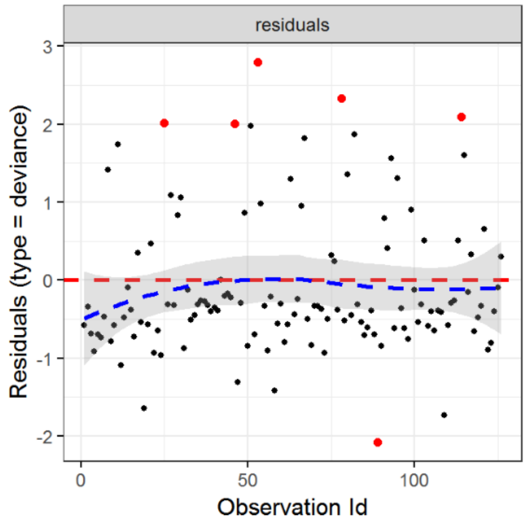

Figure S1 Migration stage cox model goodness of fit diagnoses (deviance residuals). Black dots are deviance residuals, red dots are potential outliers, red dashed line is the expected value and blue dashed line is the smoothed residuals value.

S2.2 Wintering stage

Wintering ODBA and wintering regions were selected for the cox model of the first winter period (Table S5). The results of the graphical test of the functional form using martingale residuals were also omitted here. Table S6 shows the result of assessing the proportion hazard assumption: no covariate violates the assumption. One bird which died in Morocco of electrocution was considered as potential outlier which died too earlier (Figure S2).

Table S5 Cox survival model matrix for first wintering

Matrix	coef	S.E. coef	Wald Z	p
Median winter ODBA	-1.428	0.626	-2.28	0.023*
Wintering region Europe	-2.032	0.723	-2.81	0.005*
Wintering region North Africa	-3.064	1.014	-3.02	0.003*
Model overall summary	N=76, number of events=18, df=3<br>R^2=0.202, log ratio test p=0.007*			

Table S6 Proportion hazard test for wintering model

	rho	chisq	p
Median winter ODBA	0.215	1.444	0.229
Wintering region Europe	-0.019	0.009	0.925
Wintering region North Africa	-0.289	1.237	0.266
Global	NA	6.028	0.110

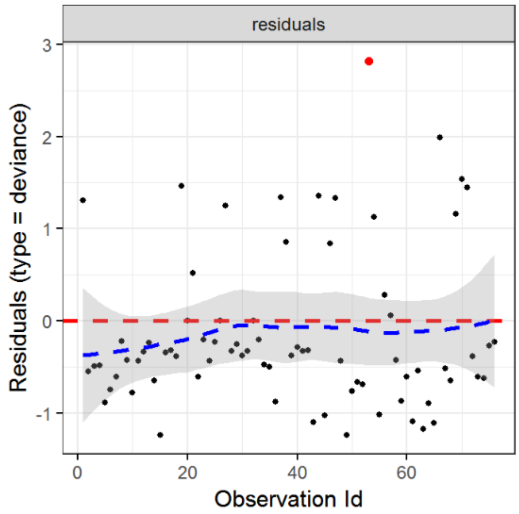

Figure S2 Winter stage cox model goodness of fit diagnoses (deviance residuals). Black dots are deviance residuals, the red dot is potential outliers, red dashed line is the expected value and blue dashed line is the smoothed residuals value.

S2.3 Sensitivity analysis

Censored survival time due to lost follow-up (vanished) or being alive at the end of study is one of the inherited features of survival data (Collett 2015). One basic assumption of survival analysis is that these cases are independent or non-formative, which telemetry studies often violate (Murray 2006). Here we had 20 storks that vanished during migration and in first winter (Fig.S3). We found some evidence of depended censoring, which suggested that vanishing time was associated with migration distance. We confirmed this by comparing the migration distance between the vanished and not vanished (those alive or with a known death time and location) individuals. These tests show that migration distance of vanished birds is significantly larger that of than alive birds (t-test, t = -3.355, df = 16.107, p= 0.004), but it is

not different from birds that died birds (t-test, t = -0.066 df = 21.472, p= 0.948).

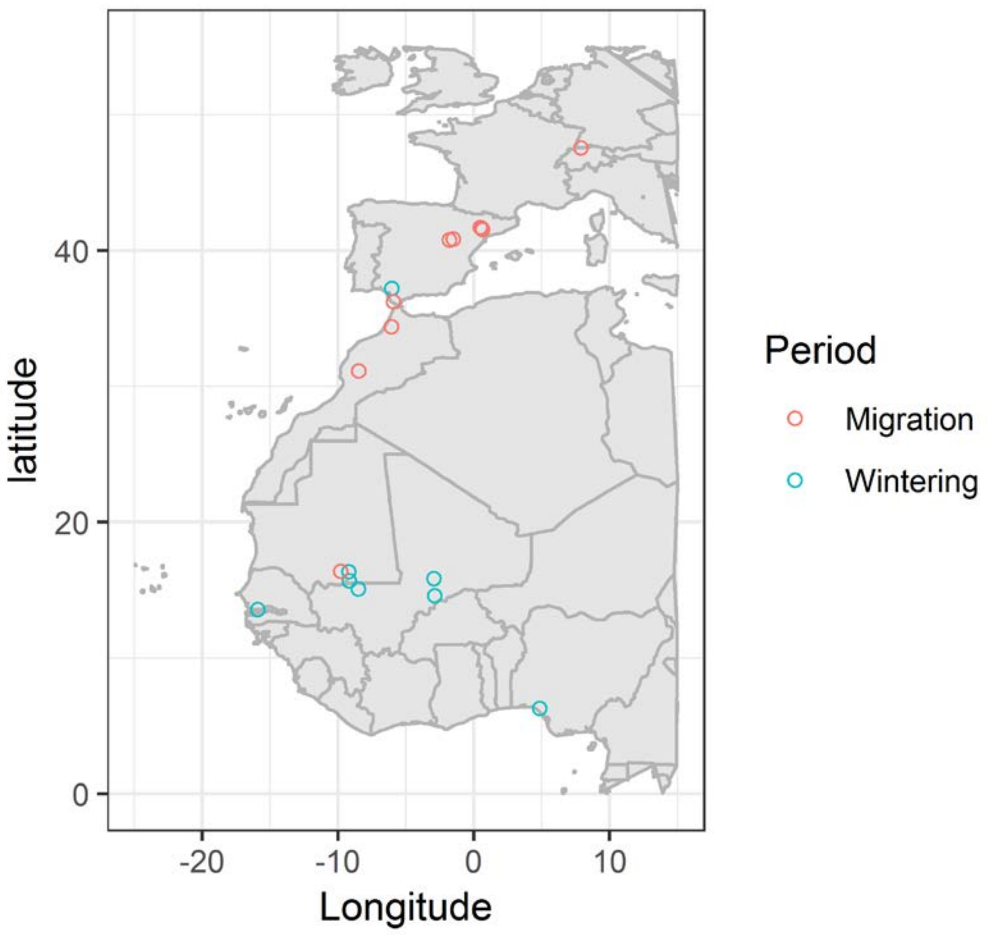

Figure S3 The GPS coordinates of the last location before individuals vanished, red open circles indicate the last coordinates before vanished during migration and blue open circles during winter.

We did not remove vanished birds or speculate on their "death time" as this will introduce artificial and unmeasurable biases as well. Instead, we tested the sensitivity of dependent censoring to determine whether it affects the results of our study (Collett 2015). We assumed that the vanished birds were at high risk of mortality and died at the time of censoring, and that they were at low risk and survived beyond the end of migration or first winter. After that, we re-fitted the two modified datasets to the models. Model matrices under high-risk and

low-risk are shown in Table S7 and Table S8. Compared to the results of the non-adjusted dataset (Table S1 and Table S5), effect sizes changed but kept their positive or negative direction, i.e. each covariate increased or deduced mortality rate in the same way. Besides wintering ODBA, all covariates remained significant, and the overall model also remained significant. Therefore, our findings are robust against dependent censoring.

Table S7 Cox Survival Model matrix for migration with vanished individuals under high vs low mortality risk.

Risk levels	High risk				Low risk			
Matrix	coef	S.E.coef	Wald Z	p	coef	S.E.coef	Wald Z	p
Fledging date	-1.743	0.798	-2.18	0.029*	-2.100	0.862	-2.44	0.015*
Fledging date’	5.216	2.312	2.26	0.024*	5.500	2.532	2.17	0.030*
Fledging date’’	-13.657	7.1009	-1.92	0.055	-13.540	7.780	-1.74	0.082
Log median migration distance	1.322	0.227	5.82	0.000*	1.344	0.247	5.44	0.000*
Median migration ODBA	-0.446	0.130	-3.44	0.000*	-0.517	0.134	-3.85	0.000*
Model overall summary	N=126, number of events=45, df=5 R^2=0.384, log ratio test p=0.000*				N=126, number of events=38, df=5 R^2=0.388, log ratio test p=0.000*			

Table S8 Cox Survival Model matrix for frist winter with vanished individuals under high vs low mortality risk.

Risk levels	High risk				Low risk			
Matrix	coef	S.E. coef	Wald Z	p	coef	S.E. coef	Wald Z	p
Median winter ODBA	-0.124	0.322	-0.39	0.699	-0.772	0.288	-2.68	0.007*
Wintering region Europe	-1.721	0.617	-2.79	0.005*	-1.866	0.727	-2.57	0.010*
Wintering region North Africa	-1.933	0.708	-2.73	0.006*	-2.952	1.050	-2.81	0.005*
Model overall summary	N=76, number of events=25, df=3 R^2=0.182, log ratio test p=0.006*				N=76, number of events=18, df=3 R^2=0.187, log ratio test p=0.010*			

Literature cited

Collett, D. 2015. Modelling survival data in medical research. Third edition. Chapman and Hall/CRC, New York. The USA.

Harrell, F. E. 2018. rms: Regression Modeling Strategies. R package version 5.1-2.

Keele, L. 2010. Proportionally difficult: Testing for nonproportional hazards in cox models. Political Analysis 18:189–205.

Klein, J. P., and M. L. Mosechberger. 2012. Survival analysis techniques for censored and truncated data. Second Edi. Springer-Verlag, New York.

Murray D. 2006. On improving telemetry-based survival estimation. Journal of Wildlife Management 70(6): 1530-1543.

Park, S., and D. J. Hendry. 2015. Reassessing Schoenfeld Residual Tests of Proportional

Hazards in Political Science Event History Analyses. American Journal of Political Science 59:1072–1087.

Therneau, T. 2015. A Package for Survival Analysis in S. version 2.38.

Xue, Y., and E. D. Schifano. 2017. Diagnostics for the Cox model. Communications for Statistical Applications and Methods 24:583–604.
